# Miscellaneous Intelligence

**Published:** 1821-06-01

**Authors:** 


### MISCELLANEOUS INTELLIGENCE.

In Kentucky, the oldest, wealthiest, and most cultivated of the Western States of America, a university has been erected which promises to be of essential benefit to that rising state. This university is situated in the town of Lexington, containing a population of about six thousand, wealthy, cultivated, and refined. This town stands in the midst of a beautiful and fertile tract of country, thirty miles in diameter, containing about ten places of worship, several public buildings, a reading room, or athaneum, and a very good circulating library. The university edifice is not surpassed in size, or elegance, by any in the United States. In the year 1820 there were 282 students of different kinds. Dr. Charles Caldwell, late of Philadelphia, is Dean of Faculty, and Professor of the Institutes—Teacher of Materia Medica, &c. with a private class in Medical Jurisprudence.

### VENTRILOQUISM.

This curious physiological phenomenon has never, perhaps, been more finely exhibited than by M. Alexandre, now performing in this metropolis, and about to proceed to different parts of the country. We entreat the attention of our professional brethren to this extrordinary operation of the respiratory organs, as well worth their notice.

### PREPARATION OF OPIUM.

We are informed by an esteemed correspondent that in the following preparation of opium the taste and smell of that medicine are completely concealed, and that it leaves much less of the unpleasant effects of opium on the system than the tincture prepared according to the pharmacopœia.

℞. Extr. Glycirrhizæ

—— Opii . . āā ℥ss.

Potass. Carb. . ʒj.

Aquæ . . . Oiij.

The whole is to be boiled to Oj. the clear liquor to be poured off, and evaporated to ℥xij. then add spirit of pimento ℥iv. cochineal in fine powder ʒss. It is said to leave no unpleasant effects on the stomach or head.

### HUNTERIAN ORATION.

Mr. Chevalier has just published his Hunterian Oration, delivered before the College of Surgeons, in February of the present year. It is written with considerable elegance and force of thought, displaying much erudition, and, what we prize more highly, philanthropy, liberality, and fine moral feeling. We shall have room but for two or three extracts from this interesting oration. The first exhibits a feature of Mr. Hunter's character, which it would be wise for all to keep before their mind's eye, from the highest to the lowest of the profession.

"He was that true and lofty spirit of science, which will not condescend to seek for eminence or wealth, by arrogating a degree of skill and dexterity that no other can attain, or vaunting a remedy with which no one else is acquainted; but which rests for its reward on the fair fame and merit of its acts; which is ever intent on the discovery of truth, and is then most of all delighted, when it can most effectually assist others in the common labour and duty of us all—the advancement of human knowledge, and the alleviation of human distress." P. 76.

After descanting, though very modestly, on the advantages which society at large has derived from the Royal Institution of Surgery, which the orator addresses, he displays his liberality of sentiment in the following passage:—

"But while we reflect with satisfaction on the prosperity of our own exertions, we must not omit to acknowledge, with the great respect that is due, the manifold public advantages which have constantly been derived from those of the Royal College of Physicians; who not only in these brighter days, but through a long æra of comparative darkness and prejudice, preserved the light of medical science unextinguished and pure; and who, for three centuries, continued to reflect honour on this nation, by members eminent for learning, great in science, and distinguished by the first excellence in professional attainments. Having enjoyed in our universities the highest means of cultivation that are provided for the human intellect, and then devoting themselves to the study and practice of medicine; these enlightened persons are most wisely and beneficially appointed the constitutional guardians of the public health; and we feel it a happiness to unite with them in our endeavours to mitigate the pains of disease; to convey consolation and hope into the chambers of anxiety and alarm; and to avert or alleviate that stroke which rends asunder all human attachments, by disuniting the conscious and immortal part of man from the world of matter, and tranferring it to the world of spirits." 93.

The following passage will afford a specimen of Mr. Chevalier's command of language as well as of vivid ideas.

"But it is not enough that we advert to the benefits derived from surgery, in the comparatively tranquil and measured course of civil life; we must not forget what it has accomplished in other and more turbulent scenes. We must turn to those seas and fields, and mantling walls, over which the thunder of the murderous cannon has roared; where fire and sword have met in awful conjunction, to support or to oppose unrelenting ambition; and where the loaded engines of war have vomited forth instant death and mutilation upon thousands and tens of thousands. How many lives have been preserved; how many days and nights of agony and torment have been prevented; what solace and consolation have been afforded in the slow and gloomy hours of anguish and suspense, by the firm and faithful hand which surgery has been enabled to stretch forth to the relief of the suffering brave! Sudden, arduous, and complicated, are often the duties, which a naval or a military surgeon is called to perform.

> Hic illi occurrit Tydeus; hic inclitus armis

> Parthenopœus, et Adrasti pallentis imago—

but well have those duties been sustained. The tried skill and humanity of our surgeons have been associated with the military glory of their country, and have divested the day of battle of half its horrors." 95.

The orator passes a high and well-merited eulogium on the character of the late, and distinguished talents of the present, President of the Royal Society; and concludes with an emphatic address to the rising generation of medical students, in which he repels the idea that our profession can have any natural tendency towards scepticism and irreligion.

"Who," says he, "can seriously contemplate the numberless operations of the principle of life, all infallibly producing their several and peculiar results, in all the different tribes of the animal and vegetable worlds—who can behold the powers with which the human body is endowed, to repair its injuries, and to relieve its diseases, as far as the destinies of our nature will permit—who can survey the demonstrations of these facts which are contained in that matchless museum, and not be compelled to unite in the glowing language of our immortal bard—

> "These are thy glorious works, Parent of Good,
> "Almighty! thine this universal frame,
> "Thus wondrous fair!" 107.

---

Some communications which the Editor has lately received have suggested the idea of his defraying, at his own expense, eight pages of closely printed letter-press, in the *Extra Limites* department of each future number of the Journal, as a bonus to the public, and a vehicle for ingenious remarks, suggestions, strictures on doctrines, practices, modes of conduct, &c. &c. in books or men, couched in general terms, and carefully devoid of personalities. These communications must be anonymous, at least to the public, and may extend from a few lines to a page; but not beyond that, if possible, least they should abridge the variety which this department is expected to produce. It is considered that a medical Argus, keeping a watchful eye on the interests and respectability of the profession, and legitimately criticising, in general terms, such deviations from the line of rectitude, propriety, truth, sound science, and morality, as may occur in men or books, must exert a very salutary influence, especially if dignity of expression and good humour be observed.

As this department is entirely at the private expence of the superintending Editor, it is requested that communications may be *post paid*, and sent as early in the quarter as possible. After the tenth of the month preceding publication day, they will be too late for that number of the Journal.

*N. B.* All appeals and defences of authors must be paid for, as usual, in the Extra Limites department.

